# Standardization Improves Discharge Care Coordination for Children with Nasogastric Tubes

**DOI:** 10.1097/pq9.0000000000000823

**Published:** 2025-07-01

**Authors:** Lisa M. Rickey, Katharine Nagle, Julia Perkins, Caroline Kohler, Benjamin Ethier, Kristen Fontaine, Susan Matherson, Anne M. Stack, Maireade E. McSweeney

**Affiliations:** From the * Division of General Pediatrics, Boston Children’s Hospital, Boston, Mass.; † Department of Pediatrics, Boston Children’s Hospital, Boston, Mass.; ‡ Department of Pediatrics, Harvard Medical School, Boston, Mass.; § Division of Gastroenterology, Hepatology, and Nutrition, Boston Children’s Hospital, Boston, Mass.; ¶ Care Management Center, Boston Children’s Hospital, Boston, Mass.

## Abstract

**Introduction:**

Discharging patients with new nasogastric tubes (NGTs) for enteral nutrition at home is complex and requires intricate care coordination and education from a multidisciplinary team. We designed a quality improvement (QI) initiative to improve efficiency and decrease variation in care coordination for patients discharged with a new NGT. Our objective was to reduce mean modified hospital length of stay (mLOS) by 10% from baseline within 6 months and sustain improvement for 12 months.

**Methods:**

Applying the Model for Improvement, we used plan-do-study-act cycles to improve NGT discharge care coordination using a multidisciplinary team. Primary interventions rooted in Lean methodology included creating a standardized discharge algorithm, utilizing nurse practitioners as care coordination champions, routine consultation of an enteral tube service (ETS), and implementing a formula substitution guide. The primary outcome measure was mean mLOS. The process measure was the time from NGT placement to ETS consult. Balancing measures were ETS consult volume and 30-day healthcare reutilization. Statistical process control charts measured the impact of interventions.

**Results:**

Baseline mLOS decreased from 8.2 to 7.4 days with a sustained reduction in process variability over time. Time from NGT placement to ETS consult decreased from 4.1 to 3.0 days. There were no changes in 30-day healthcare reutilization or ETS consult volume over time.

**Conclusions:**

A multidisciplinary quality improvement initiative effectively improved complex NGT transitional care planning and was sustained over time.

## INTRODUCTION

Nasogastric tubes (NGTs) are frequently utilized to support nutrition for hospitalized pediatric patients. NGT feeds are used for short-term (including feeding supplementation following acute illness, inadequate intake that is expected to improve) and longer-term indications (such as limited ability to feed safely by mouth due to anatomic, genetic, neurologic, and oropharyngeal conditions).^1^ Although most pediatric patients may only require NGT feeds for a limited period during hospitalization, some may require continuation of enteral nutrition via NGT at home.

Enteral tube feeding at home, which includes NGT feeding, is widely utilized and generally well tolerated.^1^ However, there are associated risks including errors in documentation and communication, equipment and devices, administration of feedings and medications, and family education and training.^2^ For example, patients and families may encounter tube malfunction and dislodgement, which introduce risk for preventable adverse events such as perforations of the gastrointestinal (GI) tract, unintentional feeding into the lung, and pneumothorax.^3^ As a result, one in ten patients with nasoenteral tubes at home may have emergency department visits within 30 days of index hospitalization for tube displacement or dysfunction.^4^ Strategies to safely transition patients requiring NGTs for home nutrition from inpatient to outpatient care and while mitigating risk for adverse events have included: engaging a multidisciplinary team in care coordination, anticipatory planning before discharge to allow adequate time for caregiver skills education, utilization of discharge readiness checklists, and identification of a post-discharge medical home.^3,5,6^

Our institution discharges hundreds of patients each year with NGTs for home use. The care coordination process is complex and requires collaboration from a large multidisciplinary team. We lacked an established, standardized pathway to guide providers through the hospital-to-home transition for this patient population, leading to unnecessarily prolonged length of stay and high variability that could introduce preventable risks for safe care delivery at home. Therefore, we designed a process and quality improvement (QI) initiative using the Model for Improvement to improve efficiency and reduce process variability in inpatient NGT care coordination. Our Specific, Measurable, Achievable, Relevant, and Time-bound aim was to reduce the mean modified hospital length of stay (mLOS) by 10% from baseline for children discharged from Pediatric Hospital Medicine (PHM) services with a new NGT within 6 months and to sustain improvement for 12 months.

## METHODS

### Context

We completed this QI project on the PHM service at Boston Children’s Hospital, a large, stand-alone quaternary care academic children’s hospital. Our PHM teams discharge approximately 3,600 patients annually and are staffed by pediatric hospitalists, residents, and nurse practitioners (NPs). Our target population included all patients discharged from PHM services from July 1, 2022, to January 31, 2024, who were prescribed enteral nutrition via NGT for home use after discharge. We included all patients discharged with a new NGT, regardless of whether feeding and nutrition were the primary indication for hospitalization, to accurately represent the patient populations we care for on our services. We excluded patients whose care coordination began on PHM but were ultimately discharged from another service and did not participate in our improvement work. However, we included patients whose initial hospital care started on a different service (eg, in an intensive care unit) but who were discharged from PHM. Patients receiving home NGT feeds at the time of hospital admission who required resumption of home services at discharge, and those with surgical gastrostomy tubes, were also excluded, because these processes are similar but distinct from our population of interest.

Our institution has had a dedicated Enteral Tube Service (ETS) since 2013, comprised of NPs and gastroenterologists who specialize in caring for patients with enteral feeding tubes (nasoenteral and surgically placed enteral tubes). This team provides consultative services and tube support for any patient started on NGT feeding, requiring assessment for more permanent gastrostomy or gastrojejunostomy tube placement, or complications from an existing enteral feeding tube. The ETS may troubleshoot tube-related complications and review feeding intolerance, provide education on tube care and management for families of children with (new) enteral tubes at home, identify and document replacement plans if the patient will be discharged home with an indwelling tube. Initiation of ETS consultation also facilitates access for patients and families to additional ambulatory Gastroenterology care services upon discharge, wherein clinic nurses triage any postdischarge NGT complications or supply needs that may arise. They also provide tube-related education to families and caregivers, and starting in September 2023, a hands-on mannequin NGT simulation teaching experience for interested patients and families.

### Intervention Planning

**Fig. 1.**
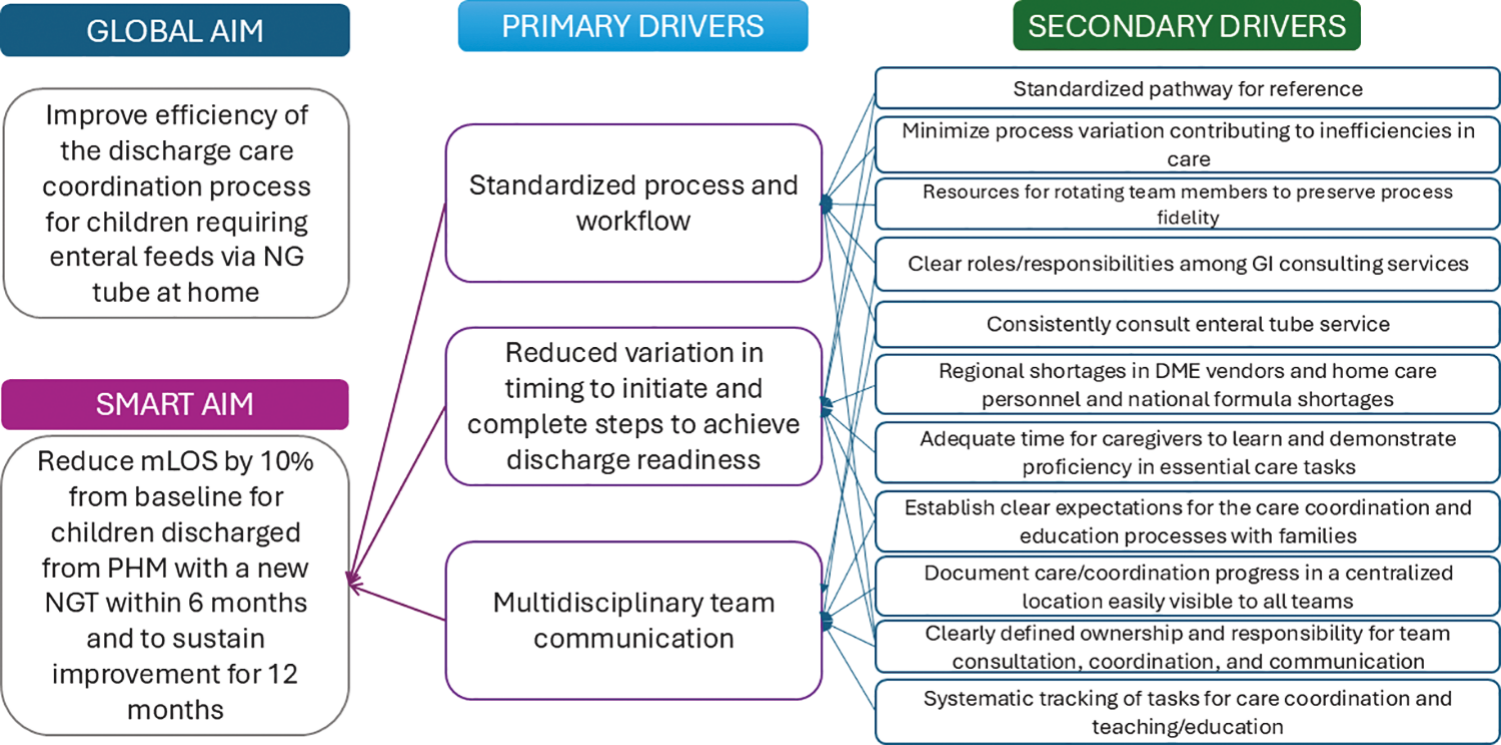
Key driver diagram for home NGT discharge optimization. Driver diagram depicting the relationship between project aim, primary drivers, secondary drivers, and associated change strategies. DME,: durable medical equipment.

We framed the project using the Model for Improvement.^7^ Our QI team assembled a multidisciplinary working group including PHM physicians and NPs, gastroenterologists and an ETS NP, dieticians, case management specialists, bedside nursing, and a parent/family representative. We used flow chart process mapping to identify the current state and understand the complexities of discharge care planning for home NGTs, including identifying process steps with high variability in execution. Following interviews with key stakeholders, front-line clinicians, and direct observations at *gemba*, the front-lines of patient care, we developed a key driver diagram with associated change strategies to guide improvement efforts (Fig. 1). We found the three main drivers to achieve our improvement aim were (1) standardized processes and workflows, (2) reduced variation in steps to achieve discharge readiness, and (3) improved multidisciplinary team communication. Our improvement team chose to focus our interventions on standardization and variability drivers. We hypothesized these interventions would have the greatest impact on improving discharges with new NGTs and would simultaneously reduce process variation and enhance team communication. In addition, we anticipated that early process standardization would guide priorities for future improvement phases.

### Interventions to Standardize the Process and Workflow

**Fig. 2.**
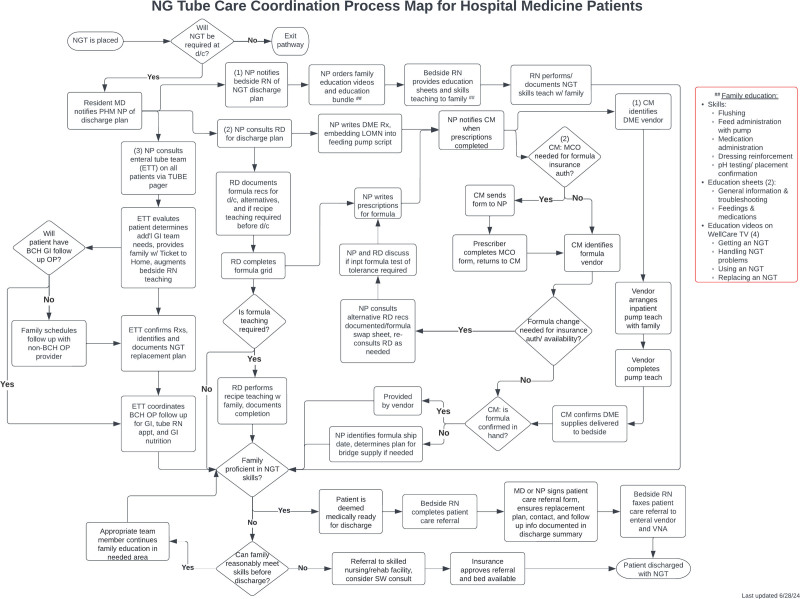
Process map. The process flow map outlines the revised workflow created during the improvement project to guide medical providers through the care coordination process for patients being discharged home with new NGTs.

Our improvement team developed a two-pronged approach to drive interventions for improvement. First, we developed a standardized workflow (Fig. 2) that could be broadly implemented on PHM services to achieve process standardization, including the following key interventions:

1. Early and universal ETS consultation for all patients being discharged with a new NGT.
2. Creation of a standardized discharge checklist of required durable medical equipment supplies and prescriptions that was accessible to all prescribers within the electronic medical record (EMR).
3. Defining and ensuring that parent/caregiver education and skills teaching were completed before discharge, with explicit task ownership.

### Interventions to Decrease Process Variation in Steps to Achieve Discharge Readiness

We integrated 2 interventions to reduce preventable delays in care associated with process variation. First, we called on principles from Lean methodology to redirect resident physicians away from the care coordination process and streamlined care coordination through team NPs.^8^ This encouraged team members to operate within their scope of practice and reduce nonutilized talent. As a result, we facilitated resident physicians focusing their efforts on direct patient care support. Second, we learned that the formula prescribing process contributed to delays. Trips to *gemba* revealed frequent and sometimes prolonged exchanges between clinicians, dieticians, and case management specialists to determine appropriate formula selections based on patient factors, availability, and insurance coverage. External factors such as regional or national formula shortages may introduce additional complexity. Therefore, with our team dietitian, we created a reference tool for acceptable formula substitutions, by age, that clinicians could easily apply without requiring routine consultation from a dietitian when necessitated by payor coverage.

### Education Campaigns

The team supported system-based interventions with an awareness campaign throughout implementation. During educational staff meetings, we provided formal education about process changes for PHM faculty, fellows, and NPs. Similarly, we provided education for nurses during unit-based staff meetings. We updated residents rotating through PHM services about the initiative and their care responsibilities during a regular case conference series at the start of their rotation. Following initial implementation, process updates for NPs, a small key stakeholder group of seven individuals, were led by the project champion on an as-needed basis throughout the initiative. As process experts, PHM NPs also provided just-in-time education for staff members on an ad hoc basis throughout the intervention.

### Concurrent Interventions

A pilot intervention to improve patient flow and capacity planning on PHM and other selected teams overlapped with implementing our interventions. This institution-driven initiative introduced an EMR-based tool to document the anticipated discharge date to improve situational awareness among clinical and patient flow teams. Later in the intervention phase, the ETS implemented a simulation program for families and caregivers to teach NGT replacement skills. Simulation sessions were offered 2–3 times per week and as needed when discharge dependent.

### Measures

The primary outcome measure was mLOS in days, defined as time from NGT placement to hospital discharge. This measure represents the first reasonable time when teams could logically and confidently begin care coordination of the NGT for home use. We defined NGT placement time as the first documented presence of the tube in the patient’s EMR (eg, in a nursing flowsheet of lines, drains, airway access). Using mLOS allowed us to control for some of the many factors that may have contributed to an individual patient’s need for a NGT. We hypothesized that a 10% reduction goal for mLOS was aspirational but feasible given the heterogeneity of medical needs in this patient population, the complexities of their care coordination, and external factors (eg, insurance authorization) that may be beyond clinical teams’ immediate control. We excluded patients with a mLOS greater than or equal to 25 days, as those patients were unlikely to have prolonged hospitalizations due to NGT or feeding-related issues alone. We estimated cost savings from our intervention by comparing room and board (direct and indirect costs) between patients in our baseline and intervention groups; data for estimated savings were obtained from our institutional business office. Process measures included time (in days) from NGT placement to ETS consult. The time-stamp for the ETS consult note in the EMR defined this. Patients who were followed by a general GI or GI subspeciality consultant (for example, aerodigestive team) did not qualify for ETS consult, and were not included in this metric, but were exposed to other aspects of the intervention bundle. The balancing measures included 30-day healthcare reutilization, defined as ambulatory visits and emergency department revisits, and frequency of ETS consultations over time.

### Data Analysis

We utilized our institution’s data warehouse platform (Microstrategy, Tysons Corner, Va.), which compiles data from our EMR for internal quality assessment and data analysis. We analyzed the impact of our interventions using statistical process control methods utilizing STATA and defined special cause variation using rules for interpreting Shewhart charts in healthcare.^9^

### Ethical Considerations

Under our institution’s policies, this project was deemed a QI initiative. It does not constitute human subjects research and therefore did not require institutional review board submission or approval.

## RESULTS

**Fig. 3.**
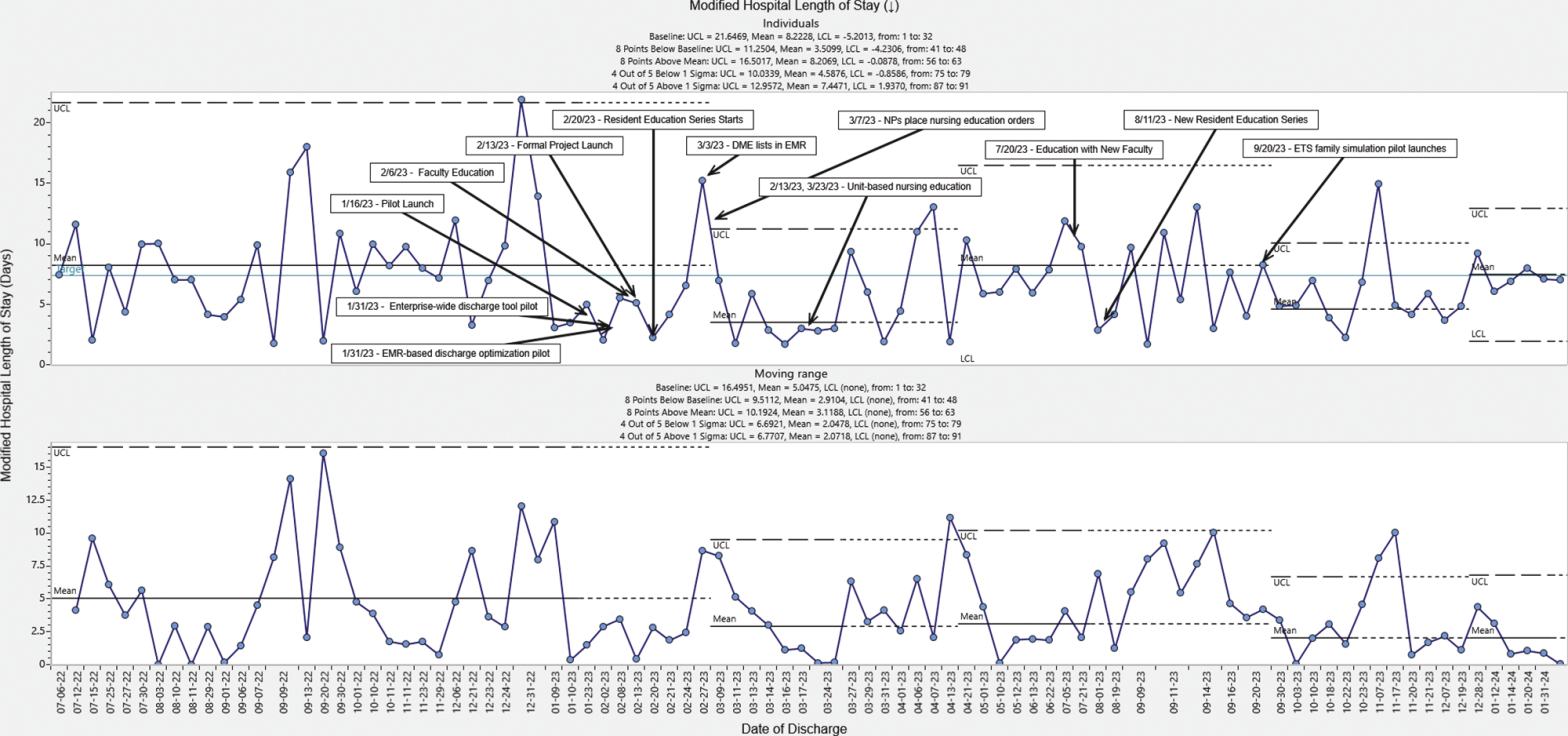
Statistical process control individuals chart (I-MR chart) of modified hospital length of stay for children discharged with NGTs for home nutrition. The I-MR chart shows a change in mLOS. Each point represents individual patient measurements in chronological order throughout the improvement project. A solid black line represents the mean centerline for mLOS. The solid blue line is the target improvement goal. Dotted lines denote the statistical upper and lower control limits. There is a downward arrow indicating the direction of intended improvement. The MR panel shows the absolute difference between the values in consecutively charted points in the control chart. The center line is the average of all MR values. LCL, lower control limit; MR, moving range; UCL, upper control limit.

**Fig. 4.**
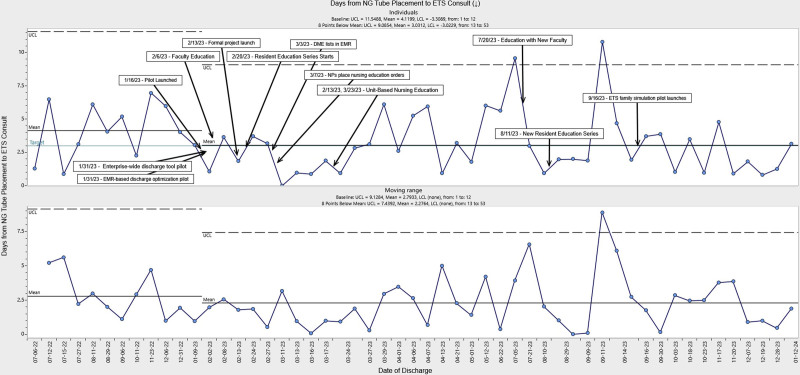
Statistical process control individuals chart (I-MR chart) of time from NGT insertion to enteral tube service consult. The I-MR chart shows a change in time from NGT insertion to enteral tube service consultation. Each point represents individual patient measurements in chronological order throughout the improvement project. A solid black line represents the mean centerline for mLOS. The solid blue line is the target improvement goal. Dotted lines denote the statistical upper and lower control limits. There is a downward arrow indicating the direction of intended improvement. The MR panel shows the absolute difference between the values in consecutively charted points in the control chart. The center line is the average of all MR values. LCL, lower control limit; MR, moving range; UCL, upper control limit.

There were 99 individual patients discharged home with a new NGT from PHM services between July 2022 and January 2024. There were 32 individuals included in the baseline period and 67 individuals in the intervention group (Fig. 3). During the first 3 months after our interventions, we observed special cause variation with the mean mLOS decreasing from a baseline of 8.2 to3.5 days. Throughout the remainder of the intervention, we observed 2 additional center line shifts due to special cause variation. By the end of the intervention study period, the average mLOS was 7.4 days, which met our target improvement goal. Despite variation in mean mLOS, we observed consistent narrowing of upper and lower control limits, indicating reduced process variability, and this change was present throughout the intervention. To quantify the value of our improvement work, we estimated average room and board cost (direct and indirect) savings resulting from our interventions, which could represent a modest decrease in average costs per patient from $44,462 to $42,273. The process measure demonstrated a reduction in time from NGT placement to ETS consult from 4.1 to 3.0 days (Fig. 4). We did not observe any nonrandom statistical changes in 30-day healthcare reutilization or in the frequency of ETS consult volume over the study period.

## DISCUSSION

Using the Model for Improvement and drawing from Lean methodology principles, we substantially decreased mLOS for children discharged home with new nasogastric tubes for enteral nutrition. By developing a process standard, providers more effectively and efficiently navigated the complex NGT discharge care coordination process, resulting in a more controlled outcome. The primary strength of this study is that we reached a consensus among a broad multidisciplinary team to develop and implement a robust framework to expedite transitional care coordination for these children and their families.

To our knowledge, this is the first improvement initiative to address care coordination for patients requiring NGTs for home nutrition before discharge. Previous studies have described the complexities of hospital-based care coordination and key elements necessary for successful transition to home enteral nutrition; however, they did not address practical ways to operationalize this transition.^5,10^ We believe that the most impactful improvements resulting from our bundled intervention were creating a standardized process for discharge care coordination and reallocating responsibilities from resident physicians to NPs, which is in line with Lean principles.^8^ Visibly mapping processes enabled team members to effectively navigate complex systems and reduce wasted time and energy, which in turn helped reduce the length of stay. Moreover, centralizing process expertise and ownership among a small, relatively stable team was fundamental to our QI success in establishing and maintaining process fidelity. It was a key driver to reduce process variability. By extension, this facilitated resident physicians to direct their attentions to the acute, time-sensitive needs of hospitalized patients and their families. There may also be a longer-term benefit to redistributing acceptable tasks to nonphysician team members, improving process efficiency and potentially reducing physician burnout while preserving quality of care.^11,12^ Finally, improved process efficiency and transparency facilitated enhanced partnership with our ETS earlier in a patient’s hospitalization, allowing more time for patient and family NGT assessment, education, and discharge planning to answer the ever-important question, “When will we be able to go home?.”

We could not compare our improvement in NGT discharge performance across similar institutions, which would have allowed a quasi-experimental approach. Medicaid data from a multi-state analysis demonstrated a median hospital length of stay of 6 days (interquartile range 3–11) for patients discharged with nasoenteral tubes.^4^ This may serve as a reference point for others seeking to identify improvement goals. We intentionally did not exclude patients or perform subgroup analyses by clinical diagnosis, medical complexity, or patient-level factors to preserve these patients’ diverse and heterogeneous nature in our improvement work. We believe all patients benefit from care standardization; however, factors such as concurrent acute or chronic medical issues, social considerations, and other care coordination needs may have contributed to the observed centerline regressions toward baseline. We intermittently audited process performance during our intervention when we observed unexpected shifts without observed changes to process fidelity. We noted a trend toward ETS consults being placed closer to the time of hospital discharge in patients with longer hospitalizations, a reasonable adaptation to ensure the care coordination and education were performed at a time most relevant to discharge. Consequently, we attribute mLOS variability over time to individual patient factors rather than a lack of intervention sustainability. Future investigation of individual patients with longer mLOS may yield additional opportunities for improvement external to the NGT coordination process. Importantly, even when the centerline shifted back toward baseline unexpectedly, the persistent narrowing of control limits supports the value of our interventions to reduce process variability.

Ultimately, more efficient discharge coordination has favorable implications for families through reduced opportunity costs and psychosocial stressors, including fewer missed days of work or school and alternative childcare arrangements. This QI work also demonstrated hospital-level value by decreasing mLOS, which improves hospital throughput, resource utilization, and care costs. We estimated modest room and board cost savings, but patient-specific factors and the heterogeneous nature of this patient population may underestimate the magnitude of these improvements.

Our study has several limitations. First, this is a single-center study, which may limit the generalizability of our results. In particular, other institutions may not have access to the ETS to provide analogous inpatient and outpatient care for patients with enteral tubes. However, the process modifications made throughout our improvement work are easily adaptable and could generate similar improvements in other contexts. Next, a hospital-wide pilot to document estimated discharge dates to improve patient flow and capacity planning was launched at a similar time to this initiative, introducing a potential confounder to our results. This concurrent intervention was unlikely to influence the care process steps most relevant to our population; however, our QI work may have benefited from the improved situational awareness for discharge planning that was available to all clinicians. In addition, we unfortunately did not have an adequate number of patients in our cohort to perform equity analyses by race/ethnicity or language in a meaningful way. Finally, the precision of our measurements may have been limited by the data available in our EMR; for example, we may underestimate the improvement in time from NGT placement to ETS consult by using consult note time-stamps in the absence of an available EMR-based order. Improvement in mLOS could have been greater because notes are customarily timestamped after evaluation.

## CONCLUSIONS

We successfully designed and implemented a QI initiative to improve the timeliness and efficiency of care for hospitalized patients requiring nasogastric tube feedings at home. We met our improvement goal for reduced mLOS and sustained improvements in reduced process variability throughout implementation. Our bundled interventions improved process fidelity and standardization, encouraged clinicians to operate in their scope, and reduced waste by removing unnecessary personnel, overprocessing, and avoidable delays in care coordination. Collaboration from a multidisciplinary improvement team was vital to drive meaningful and successful change.

## ACKNOWLEDGMENTS

The authors thank Drs. Brittany Esty and Joseph Jacobson for QI mentorship related to this work, and Mr. David Johnson for QI and data consultation. The authors acknowledge the contributions of Ms. Chava Bolotin in her role as a parent/family advisor.
